# The Role of Mast Cells in Bone Metabolism and Bone Disorders

**DOI:** 10.3389/fimmu.2020.00163

**Published:** 2020-02-07

**Authors:** Deniz Ragipoglu, Anne Dudeck, Melanie Haffner-Luntzer, Martin Voss, Jochen Kroner, Anita Ignatius, Verena Fischer

**Affiliations:** ^1^Trauma Research Center Ulm, Institute of Orthopedic Research and Biomechanics, Ulm University Medical Center, Ulm, Germany; ^2^Medical Faculty, Institute for Molecular and Clinical Immunology, Otto-von-Guericke University Magdeburg, Magdeburg, Germany

**Keywords:** mast cells, inflammation, bone disorders, osteoporosis, fracture healing

## Abstract

Mast cells (MCs) are important sensor and effector cells of the immune system that are involved in many physiological and pathological conditions. Increasing evidence suggests that they also play an important role in bone metabolism and bone disorders. MCs are located in the bone marrow and secrete a wide spectrum of mediators, which can be rapidly released upon activation of mature MCs following their differentiation in mucosal or connective tissues. Many of these mediators can exert osteocatabolic effects by promoting osteoclast formation [e.g., histamine, tumor necrosis factor (TNF), interleukin-6 (IL-6)] and/or by inhibiting osteoblast activity (e.g., IL-1, TNF). By contrast, MCs could potentially act in an osteoprotective manner by stimulating osteoblasts (e.g., transforming growth factor-β) or reducing osteoclastogenesis (e.g., IL-12, interferon-γ). Experimental studies investigating MC functions in physiological bone turnover using MC-deficient mouse lines give contradictory results, reporting delayed or increased bone turnover or no influence depending on the mouse model used. By contrast, the involvement of MCs in various pathological conditions affecting bone is evident. MCs may contribute to the pathogenesis of primary and secondary osteoporosis as well as inflammatory disorders, including rheumatoid arthritis and osteoarthritis, because increased numbers of MCs were found in patients suffering from these diseases. The clinical observations could be largely confirmed in experimental studies using MC-deficient mouse models, which also provide mechanistic insights. MCs also regulate bone healing after fracture by influencing the inflammatory response toward the fracture, vascularization, bone formation, and callus remodeling by osteoclasts. This review summarizes the current view and understanding of the role of MCs on bone in both physiological and pathological conditions.

## Introduction

Mast cells (MCs) are tissue-resident immune cells and are best known for promoting allergic reactions (1). However, research over recent decades has revealed important functions of MCs in numerous physiological conditions, including the regulation of angiogenesis and tissue homeostasis, but also in pathological conditions, such as gastrointestinal and cardiovascular diseases. MCs are distributed throughout several tissues, including the skeletal system (2). They are suitable candidates to be involved in bone metabolism and bone disorders, because MCs store and de novo synthesize many mediators, including cytokines and enzymes (3), which have been shown to regulate bone homeostasis and to be involved in the pathogenesis of several skeletal diseases (4). Indeed, increased numbers of MCs have been found in patients with reduced bone mass observed in mastocytosis or postmenopausal osteoporosis (5, 6). Furthermore, it has been shown that the synovial fluids of patients suffering from rheumatoid arthritis (RA) or osteoarthritis (OA) contain increased MC numbers and elevated concentrations of certain MC mediators including tryptase or histamine (7, 8). Importantly, numerous experimental studies using MC-deficient mouse models confirmed the involvement of MCs in the pathologies of osteoporosis and arthritis (9, 10). Interestingly, several groups using MC-deficient mouse models discovered that MCs also play an important role in the process of bone fracture healing and might be involved in the regulation of osteoclastogenesis (10, 11). However, further research needs to elucidate the molecular mechanisms of MC actions in these various physiological and pathological conditions.

The scope of this review is to provide an overview of the physiological role of MCs in bone homeostasis based on the current state of knowledge. Moreover, the role of MCs in bone disorders will be discussed, focusing on osteoporosis and bone fracture healing, including both current clinical and experimental data. The involvement of MCs in RA and OA will be discussed only briefly, because there are several comprehensive reviews from other authors, which summarize the important function of MCs in these bone disorders (12–14).

## Mast Cells and Their Physiological Roles

MCs are tissue-resident hematopoietic cells and are identified by their large number of secretory granules, which contain a broad variety of preformed mediators, including biogenic amines (e.g., histamine), heparin, cytokines [e.g., tumor necrosis factor (TNF), interleukin-6 (IL-6)], enzymes (e.g., chymases, tryptases), and various growth factors [e.g., vascular endothelial growth factor (VEGF), fibroblast growth factor (FGF)] (3). Unlike most other hematopoietic cells, mature MCs are not found in the circulation under physiological conditions. They are released from the bone marrow as MC progenitors (MCps). MCps are characterized by their expression of CD34, as are other early hematopoietic cells, and by MC-related surface markers, including CD117 (c-Kit), also known as stem cell factor (SCF) receptor (15). c-Kit is highly expressed on hematopoietic stem cells and its activity is crucial for hematopoiesis. Interestingly, only MCs retain c-Kit expression throughout their lifetime, whereas it is lost in other hematopoietic lineages during differentiation. SCF/c-Kit signaling is essential for MC growth, differentiation, and survival. Late MCps also express the high-affinity immunoglobulin E (IgE) receptor (FcεRI), as do mature MCs, however, MCps are less or non-granulated in contrast to mature MCs, which have many metachromatic granules (15–17). Committed MCps enter the target tissues and complete their maturation based on the local microenvironment (18). That is why their types and amounts of mediators can vary during MC maturation depending on the respective tissue (19). While MCs are located in almost all tissues, high numbers are found in tissues facing the external environment, including the skin, lungs, and intestines, where pathogen exposure is most likely. Thereby, MCs serve as immunological sentinels in the first line of defense. Their long lifespan of up to several months as well as their perivascular, perilymphatic, and perineuronal locations potentiate MCs to respond rapidly to pathogens. Moreover, they can also react to humoral and neuronal stimuli as well as tissue damage (e.g., physical injury inducing damage-associated molecular patterns) or environmental insults (20, 21).

Mature MCs are mainly divided into two subsets in both humans and rodents, which differ in their anatomical distribution and the types of proteases produced (22). In humans, so-called MC_T_ express only tryptases and are located predominantly in the lungs and small intestinal mucosa, whereas MC_TC_ produce both tryptases and chymases as well as carboxypeptidase A3 (Cpa3). MC_TC_ predominate in the skin and the submucosa of the small intestine (23). In rodents, MCs are classified into connective tissue MCs (CTMCs) and mucosal MCs (MMCs). In terms of tissue localization and protease content, CTMCs are thought to resemble human MC_TC_, whereas MMCs closely correspond to human MC_T_. CTMCs are particularly located in the skin, peritoneal cavity, and submucosa of the intestine, while MMCs occupy the mucosal epithelium of the lungs and the gastrointestinal tract (24). Both MC subtypes are mainly identified via their protease content. While MMCs predominantly express the chymases MC protease-1 (Mcpt-1) and Mcpt-2, CTMCs express the chymases Mcpt-4 and Mcpt-5 as well as the tryptases Mcpt-6 and Mcpt-7, and additionally Cpa3. Furthermore, both subclasses react differently in response to stimulation and inhibition by drugs and interactions with T cells. MMCs expand remarkably during T cell-dependent immune responses, whereas CTMCs do not require T cells for expansion (22–24).

MCs can be activated by numerous factors, including immunoglobulins, cytokines, neuropeptides, complement proteins, and pathogen-associated molecular patterns (e.g., by alarmins). Activation results in the release of preformed and newly synthesized mediators via degranulation. Furthermore, depending on the stimulus, MCs can also release the mediators selectively without degranulation (25). The most important and well known mechanism of MC activation is the crosslinking of the FcεRI via IgE and multivalent antigen complexes (26). FcεRI crosslinking triggers a cascade of intracellular signaling events, comprising protein phosphorylation, intracellular calcium mobilization, and transcription factor activation, and culminates in MC degranulation (27). Because MCs are present at the tissue boundaries, they are the first immune cells encountering invading endogenous and exogenous pathogens. Thereby, MCs can be activated directly by pathogens as well as by many pathogen-derived soluble products, including lipopolysaccharide (derived from gram-negative bacteria) and peptidoglycan (derived from gram-positive bacteria). They directly activate MCs via toll-like receptors (TLRs) or indirectly by activating the complement system through its receptors on MCs. Activation through TLRs induces selective cytokine synthesis and release depending on the stimuli, allowing specific responses to certain immunological insults (21, 25, 28, 29). For example, whereas TLR1 stimulation results in degranulation and additional IL-1 production, TLR2 activation induces the synthesis of cytokines and leukotrienes without degranulation (30). MCs can directly kill the pathogens by phagocytosis or extracellular traps similar to neutrophils. Additionally, they enhance the mucus production of epithelial cells to immobilize pathogens and modulate vascular permeability and blood flow to initiate rapid immune cell recruitment of effector cells, including neutrophils, eosinophils, and natural killer cells. Therefore, MCs play an important role in initiating the immune response. MCs and their products are also involved in the regulation of adaptive immune responses. For example, they modulate the migration, maturation, and activation of dendritic cells, present antigens to cytotoxic T cells, and attract effector T cells through their mediators (2, 21, 27, 31).

Beyond the host defense, MCs have many physiological functions. Several studies demonstrated that MCs enhance angiogenesis by secreting pro-angiogenic factors, including VEGF, basic FGF, TNF-α, heparin, histamine, IL-8, and various proteases (32, 33). Furthermore, MCs are considered important for tissue homeostasis, because many of their mediators, including FGF, histamine, and tryptase, induce epithelial cell and fibroblast proliferation. In addition, MCs are the main source of proteases, including tryptases, chymases, and cathepsin G, which activate matrix metalloproteinases (MMPs), thus initiating tissue remodeling (28, 34). MCs also appear to be critical for wound healing. They are present in the connective tissue of the skin in large numbers and are activated by injuries caused by trauma, heat, irradiation, or chemical agents. Thereby, MCs influence the inflammatory response, revascularization, and tissue formation and remodeling (35, 36). However, experimental studies are contradictory as to whether or not MCs promote skin wound healing. These contradictions might depend on the type and size of the wound and the mouse models used (37–39).

## The Role of Mast Cells in Physiological Bone Turnover

**Figure 1:**
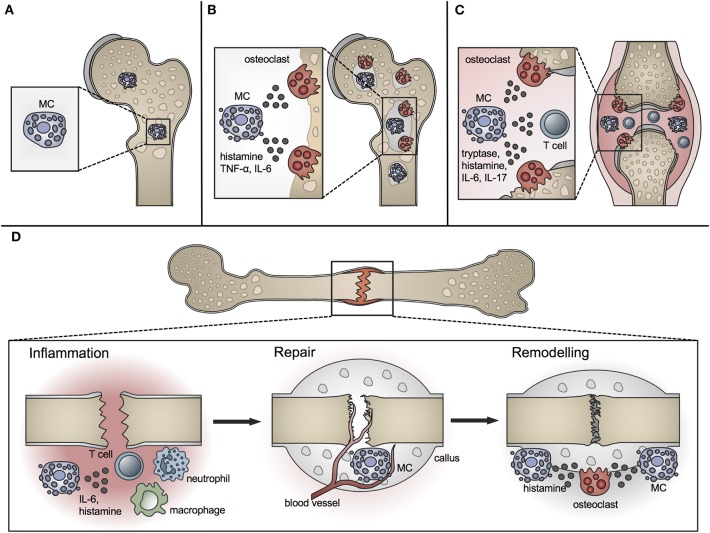
Role of MCs in physiological bone turnover and bone disease. **(A)** In physiological bone turnover, few MCs are located in the bone marrow of the metaphysis, preferentially adjacent to bone surfaces. **(B)** In osteoporotic bone, more MCs are found in the bone marrow which are frequently co-localized with osteoclasts and influence their resorption activity by releasing mediators including histamine, TNF-α and IL-6. **(C)** In rheumatoid arthritis, increased MC numbers and concentrations of MC-mediators including histamine, tryptase, IL-6, and IL-17 are found in the inflamed joint, inducing osteoclastic bone resorption and T-cell driven inflammation. **(D)** In fracture healing, MCs regulate bone-fracture induced inflammation by releasing inflammatory cytokines including IL-6, and influence innate immune cell recruitment. During the repair phase, few MCs are located in the fracture callus mainly near blood vessels; MC numbers increase during callus remodeling, where MCs are found in close proximity to osteoclasts and regulate bone resorption by releasing osteocatabolic mediators including histamine.

A role for MCs in bone metabolism was long suspected (40). Whereas MCs are located in low numbers in the bone marrow at the epiphysis and diaphysis, they are numerous in the metaphyseal bone marrow, where bone remodeling mainly occurs (41). They are preferentially located adjacent to bone surfaces undergoing bone growth or turnover (Figure 1A). MCs at the endocortical bone surface are more flattened compared to those at a distance from the bone surface, which are typically round shaped (42). Their close proximity to the bone remodeling surface and the wide spectrum of their mediators, including histamine, heparin, proteases, and various cytokines, raise the question of a potential role for MCs in bone physiology. Many of the MC mediators are able to induce or modulate osteocatabolic effects by promoting osteoclastogenesis (e.g., histamine, TNF, IL-6) and/or inhibiting osteoblast activity (e.g., IL-1, TNF) (4, 43). By contrast, other mediators could act in an osteoprotective manner by stimulating osteoblasts [e.g., transforming growth factor-β (TGF-β)] or reducing osteoclastogenesis [e.g., IL-12, interferon-γ (IFN-γ)] under certain circumstances (4). Table 1 summarizes the proposed or proven roles of MC mediators in bone formation and resorption.

**Table 1:** Selected MC mediators with effects on bone formation and bone resorption.

**Mediators**	**Bone formation by osteoblasts**	**Bone resorption by osteoclasts**
**PRE-FORMED**		
Amines		
Histamine	↑ Bone formation after depleting the histamine-producing enzyme (44)	↑ Osteoclast formation and bone resorption (43, 45)
		↓ Osteoclast activity and recruitment after histamine blocking (46, 47)
Serotonin	↓ Osteoblast formation (48)	
Enzymes		
Chymase	↑ Bone formation in Mcpt4-deficient mice (49)	
Proteoglycans		
Heparin	↓ Osteoblast formation (50, 51)	↑ Osteoclast formation and bone resorption (52)
Chemokines		
IL-8	↑ Bone formation (53)	↑ Osteoclast formation and bone resorption (54, 55)
MCP-1	↓ Bone formation in MCP-1-deficient mice (56)	↓ Osteoclast formation in MCP-1-deficient mice (57)
Polypeptide		
Renin		↓ Bone resorption after renin inhibition (58)
Substance P	↑ Osteoblast and bone formation (59–61) ↓ Bone formation in the absence of substance P (62)	
Glycoproteins		
Osteopontin		↓ Osteoclast formation and bone resorption in osteopontin-deficient mice (63, 64)
***De novo***		
Cytokines		
IL-1		↑ Osteoclast formation and bone resorption (65, 66)
IL-1β	↑ Osteoblast formation (67)	↓ Osteoclast formation (68)
IL-4		↓ Osteoclast formation and bone resorption (69–71)
IL-6	↓ Bone formation in IL-6-deficient mice (72) ↑ Bone formation (73, 74)	↑ Osteoclast formation and bone resorption (75, 76)
IL-10	↓ Bone formation in IL-10-deficient mice (77)	↓ Bone resorption (78)
IL-11		↑ Osteoclast formation (79, 80)
IL-13		↓ Osteoclast formation and bone resorption (69)
IL-15	↓ Osteoblast apoptosis (81)	↑ Osteoclast formation (82)
IL-18	↑ Bone formation (83)	↑ Osteoclast formation (84) ↓ Bone resorption (85)
IFN-γ		↑ Osteoclast formation and bone resorption (86) ↓ Osteoclast formation (87–89)
MIP-1α		↑ Osteoclast formation (90)
TNF-α	↓ Osteoblast formation (91)	↑ osteoclast formation and bone resorption (92–94)
TGF-β	↑ Osteoblast and bone formation (95, 96)	↓ Osteoclast formation (97)
Phospholipid metabolites		
Prostaglandin E2	↑ Osteoclast formation and bone resorption (98)	
PAF		↓ Bone resorption in PAF receptor-deficient mice (99)
Growth factors		
FGF	↓ Bone formation in FGF-2-deficient and -overexpressing mice (100, 101)	
GM-CSF	↑ Osteoblast formation (102)	↑ Osteoclast formation (103) ↓ Osteoclast formation (104)
M-CSF		↑ Osteoclast formation (105, 106)
SCF		↑ Osteoclast formation (107)
Nitric oxide		
NO	↓ Bone formation in NO synthase-deficient mice (108)	

To investigate the role of MCs and MC-derived products, ideally MCs would be selectively inhibited with a compound or depleted by genetic modification. Because there are no human conditions with reduced numbers or a complete absence of MCs, most data concerning the physiological role of MCs in bone development and turnover were gained either *in vitro* or in MC-deficient mouse models. To date, MC-deficient mice with mutations in the c-Kit receptor (Kit^W/W−v^ and Kit^W−sh/W−sh^ mice) or its ligand SCF (Kitl^Sl/Sl−d^ mice) have been widely used. Whereas the point mutation KitW prevents cell surface c-Kit expression, the KitW-v mutation reduces the receptor kinase activity. KitW-sh is an inversion mutation and affects the transcriptional regulatory elements at the c-Kit transcription site. Furthermore, several mutations of the SCF ligand, including KitlSl and KitlSl-d, lead to a complete or partial deletion of the SCF gene. Because SCF/c-Kit signaling is essential for MC growth and survival, mice with alterations in this signaling lack MCs (109, 110).

Silberstein et al. using Kit^W/W−v^ mice were the first to suggest that MCs might play a role in physiological bone turnover. They found that bone remodeling is delayed in this mouse model because of reduced osteoclast recruitment and osteoblast activity (111). Other studies in Kit^W−sh/W−sh^ mice reported an osteopenic bone phenotype with high bone turnover because osteoclast activity exceeds osteoblast activity (112–114). However, c-Kit-dependent MC-deficient mouse models have many other abnormalities, because c-Kit is expressed not only by MCs but also by numerous other cells, including hematopoietic progenitor cells. Importantly, c-Kit is essential for osteoclast development and also regulates osteoblast activity (113, 115). Therefore, it is difficult to distinguish the effect of MCs on bone physiology from pleiotropic c-Kit effects in these mouse models.

In contrast to the above-mentioned studies, our group proposed that MCs do not affect bone formation and turnover under physiological conditions (10). We used the Mcpt-5 Cre R-DTA mouse line, a c-Kit-independent model of MC deficiency expressing diphtheria toxin (DT) under the control of the Mcpt-5 chymase promoter, which is specific for CTMCs and drives Cre-specific ablation of these cells (116). It has been previously proven that these mice specifically lack CTMCs, whereas other immune cell populations are unaffected. Mcpt-5 Cre R-DTA mice have been demonstrated to be useful in elucidating the function of MCs in immune disorders, including contact allergy and RA (9, 116, 117). Therefore, our study may more reliably reflect the role of MCs in bone metabolism than previous investigations (10). We analyzed the bone phenotype in young and adult female and male Mcpt-5 Cre R-DTA mice and compared it to MC-competent mice. The size and shape of the skeleton were not different. We could not detect significant alterations in bone microstructure or osteoblast and osteoclast numbers or activities in MC-deficient mice compared to age- and sex-matched wildtype mice. Bone mass decreased with aging, particularly in the trabecular compartment, both in MC-competent and -deficient mice. These results suggest that MCs might be redundant for physiological bone turnover as well as in age-induced bone loss (Figure 1A) (10). However, further studies are needed to elucidate the function of MCs in bone homeostasis. Thereby, other c-Kit-independent mouse models could be used, which were developed to overcome the abnormalities related to c-Kit structure or expression. Feyerabend et al. generated Cpa3^Cre/+^ “Cre-Master” mice by using a knock-in strategy to induce Cre expression under the control of the MC Cpa3 promoter, which yielded deletion of CTMCs and MMCs by a genotoxic Trp53-dependent mechanism. However, Cpa3 is also expressed in basophils, therefore, Cre-Master mice had slightly altered basophil numbers (118). Likewise, another constitutive MC-deficient mouse model Cpa3-Cre; Mcl-1^fl/fl^ was generated by crossing Cpa3-Cre transgenic mice with mice having a floxed allele of myeloid cell leukemia sequence 1 (Mcl-1). Cpa3-Cre; Mcl-1^fl/fl^ mice are deficient in both CTMCs and MMCs, but also exhibit reduced basophil numbers (119). In addition to constitutive MC-deficient mouse models, some inducible models are also available. Transgenic Mas-TRECK mice (for Mast cell-specific enhancer mediated Toxin Receptor mediated Conditional cell Knock out) express the human DT receptor (DTR) under the control of an intronic enhancer element of the IL-4 gene, which is essential for IL-4 expression in MCs but not in other immune cells. Repeated intraperitoneal DT injection results in complete MC deletion accompanied by transient blood basophil depletion (120, 121). Recently, Dahdah et al. generated a knock-in mouse model called RMB (Red Mast Cell and Basophil) mice (122). The FcεRI β chain of these mice includes a cassette composed of a sequence coding for the bright red td-Tomato (tdT) fluorescent protein and human DTR, allowing both visualization and conditional ablation of MCs and basophils. Although both basophils and MCs were deleted after DT injections, the authors reported that basophils were fully reconstituted 12 days after DT treatment, whereas MCs remained absent (122). However, to the best of our knowledge, in none of these mouse models have to date the skeletal phenotype or bone turnover been analyzed.

In conclusion, the experimental data regarding a possible regulatory role for MCs in physiological bone turnover are contradictory and dependent on the mouse model used. Therefore, further translational studies on human MCs with respect to their role in bone metabolism are needed. There are no human MC-deficient conditions. However, patients with mastocytosis who display abnormal high MC numbers often suffer from bone disorders (see section Osteoporosis). Analyses of the bone turnover of these patients over their course of treatment (mainly anti-histamine treatment) could provide further insights into MC functions in human bone. Complementary, *in vitro* studies including co-culture models of human MCs derived from healthy individuals and mastocytosis patients and human osteoblasts and osteoclasts could provide deeper mechanistic insights into the interaction of MCs and bone cells in humans.

## The Role of Mast Cells in Bone Disorders

### Osteoporosis

Osteoporosis is a major bone disorder, which is characterized by the deterioration of bone microarchitecture and bone mass reduction. This results from an imbalanced activity of osteoblasts and osteoclasts and leads to an increased fracture risk (123). In Europe, ~20 million people suffer from osteoporosis with an annual incidence of 2.7 million fragility fractures (124). Osteoporosis is categorized into primary and secondary forms. Primary osteoporosis is the most common form, including postmenopausal osteoporosis, which results from a decline in sex hormone levels, and age-related osteoporosis, which gradually develops during aging. Secondary osteoporosis is caused, for example, by drugs (e.g., corticosteroids, barbiturates), comorbidities (e.g., kidney diseases, diabetes, hyperparathyroidism, mastocytosis), and adverse lifestyle and nutrition (e.g., cigarette smoking, alcohol abuse, immobilization, malnutrition) (123, 125). Despite extensive research during recent decades, the pathomechanisms of osteoporosis are still not completely understood. There is evidence that MCs contribute to this multifactorial disease, because increased MC numbers have been found in individuals with bone loss (Figure 1B) (5, 6, 40, 126, 127).

Postmenopausal osteoporosis is driven by the decline of estrogen after menopause, which induces increased bone resorption by osteoclasts (128). In 1983, Fallon et al. reported increased numbers of MCs in iliac crest biopsies of females with postmenopausal osteoporosis compared to non-osteoporotic males and females, indicating an involvement of MCs in bone loss (5). Confirming this, other authors similarly observed MC accumulation in bone biopsies of osteoporotic patients compared to healthy controls (6). Interestingly, treatment of postmenopausal females with calcium and promethazine, a blocker of the histamine H1 receptor, significantly increased bone mineral density compared to calcium treatment alone (129). This indicates that histamine, one of the main preformed components in MC granules, could be involved in osteoporotic bone loss (Figure 1B). Experimental studies in ovariectomized (OVX) rodents, a common experimental model for postmenopausal bone loss, confirmed the clinical observations. Lesclous and Saffar demonstrated that after OVX-induced estrogen decline the MC numbers in the rat bone marrow were significantly increased (130). The authors further showed that the accumulation of MCs started early and was associated with the increase in osteoclast numbers induced by OVX (131). Confirming this, our group showed that Mcpt-5 Cre R-DTA mice, which lack CTMCs, were protected from OVX-induced bone loss and no increase in osteoclast numbers or activity occurred after OVX (10). Using Mcpt-5 Cre tdRFP MC reporter mice, we further found that after OVX the MCs, and osteoclasts were not only enhanced in number but also frequently co-localized (10). These results are strong indications that MCs may promote osteoclast formation under estrogen-deficient conditions (Figure 1B). Confirming this, further *in vitro* studies investigating osteoclast formation under the influence of MC supernatants revealed that estrogen strongly affects MCs and their mediator release (10). When estrogen was present, supernatants derived from MCs that were stimulated with the complement anaphylatoxin C5a, an inducer of MC degranulation, did not enhance osteoclast formation *in vitro* in a preosteoclastic cell line (RAW 264.7 cells) nor in primary bone marrow-derived osteoclast precursors. By contrast, when estrogen was absent, osteoclast formation was induced, suggesting that estrogen has an inhibitory effect on the osteoclast-inducing potential of MCs (10). Indeed, estrogen receptors (ER) are expressed in MCs of various tissues (132, 133) and several groups reported that estrogen influences MC migration, degranulation, and cytokine release (133–138). However, the observed effects are not always consistent. Some authors found that estrogen induces MC degranulation (134, 135), whereas others reported inhibitory effects on the mediator release (136, 137). For example, estrogen did not stimulate the degranulation of MCs derived from ERα knockout mice (134), indicating that estrogen is involved in MC activation via ERα signaling. In agreement with this, estrogen treatment of the human MC line HMC-1 induced the de novo production of tryptase β1 and MC degranulation (135). By contrast, OVX-induced estrogen deficiency reduced MC degranulation in the rat mammary gland (137), and Kim et al. showed that estrogen treatment diminished the *in vitro* release of MC cytokines, including TNF-α and IL-6 (136). Therefore, the effects of estrogen on MC degranulation and mediator release appear to depend on the tissue investigated and the experimental model used.

MCs may also play a role in the development of age-related osteoporosis, the second type of primary osteoporosis, because Frame and Nixon already in 1968 described that MC numbers were increased in bone marrow aspirates of aged female and male patients with reduced bone mineral density compared to healthy controls (126). Because both males and females are affected by age-related osteoporosis, MCs appear to also provoke estrogen-independent osteoclastogenic effects. This is supported by the observation that MCs also play a role in secondary forms of osteoporosis. Most of these indications arise from mastocytosis, a disease characterized by abnormally high MC numbers in one or more organ (139). The clinical picture is categorized into cutaneous mastocytosis, which is restricted to the skin, and systemic mastocytosis (SM), where high MC numbers infiltrate the skin and/or one internal organ, for example, the gastrointestinal tract, bone marrow, lymph nodes, liver, and spleen (139). Mastocytosis is caused by gain-of-function point mutations within the SCF/c-Kit signaling axis, most prominently D816V (140), resulting in a constitutively active c-Kit receptor. This leads to increased MC proliferation, maturation, survival, and activity (141). The boost of released mediators, mainly of histamine and pro-inflammatory cytokines, and excessive MC infiltration cause mild to severe organ-specific symptoms, including flushing, syncope, anaphylactic shock, diarrhea, vomiting, ascites, and hypertension. The clinical picture of SM is very heterogeneous, ranging from indolent to aggressive forms with severe organ dysfunctions (139). Of note, ~50% of patients display a skeletal involvement (142). Several case studies and small clinical trials described a reduced bone mass and an increased fracture occurrence in patients with MC accumulation in the bone marrow (143–146). Larger cohort studies confirmed the high prevalence of osteoporosis (up to ~60%), and fragility fractures (up to ~40%) in SM patients (147–150), as recently reviewed in more detail by Greene et al. (151). The pathomechanisms of MC-induced bone loss are not yet fully understood. Seitz et al. found increased osteoblast and osteoclast numbers in patients with indolent SM, indicating a high bone turnover status (152). Confirming this, bone formation and resorption markers were found to be increased in SM patients (153). However, other authors reported increased serum levels of dickkopf 1 and sclerostin, both inhibitors of the osteoanabolic Wnt signaling pathway, indicating reduced bone formation (154, 155). IL-6 levels are also increased in SM, and correlate with the severity of the symptoms and bone loss (155, 156). The existing data on tryptase levels, a marker for MC activity, in SM are inconsistent. Many authors describe increased concentrations, which correlate with reduced bone mass, whereas others report normal levels despite bone loss (148, 150, 155).

MCs appear also to be involved in secondary bone loss induced by malnutrition or immobilization. Urist et al. observed an accumulation of MCs in osteoporotic bones of rats fed a calcium-deficient diet (40). Additionally, in bone loss caused by the unloading of the hind-limbs in rats, MC numbers were significantly increased (127). These results indicate that MCs may regulate osteoclast activity independently from endocrine dysregulation or inflammatory stimuli. Confirming this, male patients suffering from idiopathic osteoporosis also displayed higher numbers of MCs that were highly organized in clusters in biopsies of the bone marrow. In these patients, the urine N-methylhistamine concentration, a marker for increased MC activity, correlated with the reduced bone mineral density (157).

The above-mentioned clinical and experimental studies suggest that MCs may be involved in osteoporosis development by promoting osteoclast formation (Figure 1B). The question arises as to which MC mediators are mainly responsible for the observed osteoclast-stimulating effects. There are many possible candidates, including histamine, heparin, TNF, IL-6, and receptor activator of nuclear factor kappa B ligand (RANKL), as listed in Table 1. One of the main components in preformed MC granules is histamine, which was already shown to be associated with bone resorption in RA (158). Furthermore, in patients with SM, histamine levels were reported to predict osteoporotic manifestations (144, 147). Confirming this, histamine-deficient mice displayed an increased bone mass because of reduced osteoclast numbers and were also completely protected from OVX-induced bone loss (44). Lesclous et al. injected histamine receptor blockers in OVX rats, which prevented the OVX-induced bone loss by reducing osteoclast numbers (45, 159). In the above-mentioned study of our group (10), osteoclast formation and activity were studied *in vitro* in the presence of supernatants harvested from MC cultures stimulated with C5a (induces the release of preformed granule-stored mediators). Notably, the blockade of the histamine H1 receptor abolished osteoclast formation by MC supernatants, indicating that histamine may play a crucial role in MC-mediated osteoclast activity (10). This is confirmed by the already above-mentioned clinical study, which demonstrated that the blockade of the histamine H1 receptor with promethazine significantly increased bone mineral density in postmenopausal women (129). However, our experimental data also showed that histamine alone supported osteoclast formation but not their resorption activity (10). This indicates that histamine is not the only MC-derived factor involved in osteoclast activation and that MC-osteoclast interaction might be much more complex.

### Rheumatoid Arthritis

RA is a systemic autoimmune disease affecting around 1% of the population, which is associated with a chronic joint inflammation (160, 161). The inflamed joint is characterized by a massive infiltration of immune cells, extensive hyperplasia of synovial macrophages and fibroblasts and thickening of the synovial membrane. The unrestrained inflammatory response leads to the formation of an invasive structure, calles synovial pannus, which finally causes cartilage destruction and bone erosions. The clinical picture is characterized by swelling, pain, and stiffness of the affected joints (160). The pathogenesis of RA is complex and still not entirely known. In addition to other immune cell populations, MCs have been suggested to play a crucial role, because MCs are abundant in inflamed synovial joints of RA patients, especially around blood vessels in the synovial sub-lining, at the cartilage-pannus junction at sites of cartilage erosions, and in joint fluid (Figure 1C) [comprehensively reviewed by Rivellese et al. (162)]. Importantly, some of the clinical studies observed a correlation of MC numbers with joint inflammation and disease activity (163–167). In addition, the levels of MC mediators, including histamine and tryptase, were significantly increased in the synovial tissue of RA patients (Figure 1C) (8, 168, 169). Therefore, these studies support MC involvement in the pathogenesis of RA.

In agreement with this, MCs are highly responsive to the inflammatory milieu in the synovial joint. For example, they are stimulated by IL-33 and IL-6 (170, 171). Moreover, it has been shown that synovial MCs can be activated by immune complexes, auto-antibodies and complement factors as well as by direct cross-linking of Fc-receptors (172–175). MCs might contribute to the pathogenesis of RA by different mechanisms, which were reviewed in detail by other authors (12–14). Briefly, synovial MCs can rapidly release and produce inflammatory cytokines and chemokines and thus contribute to joint inflammation and immune cell recruitment. For example, MC-derived IL-1 is involved in the initiation of autoantibody-mediated arthritis (176), and activated MCs in human synovial tissue produce TNF-α, IL-1β, and IL-1 receptor antagonist (177). Additionally, MC-derived proteases may play important roles in cartilage and bone breakdown. Histological analysis of inflamed joint specimens showed abundant MC tryptase present in areas of cartilage destruction (178). Supporting these findings, mice deficient in Mcpt-6 or Mcpt-7 displayed an attenuated disease activity and reduced bone and cartilage destruction (179). Similarly, mice lacking the MC chymase Mcpt-4, showed a reduced joint inflammation and pannus formation, diminished cartilage destruction probably due to a reduction in MMP-2 and MMP-9 (180). Of note, the joints of Mcpt-4 deficient mice displayed less infiltrates of MCs and mononuclear cells implicating a crucial role of this MC chymase in disease progression (180). Importantly, some MC mediators, including histamine, TNF-α, IL-6, IL-11, and IFN-γ, have the capacity to increase osteoclast activity (see Table 1), and thus may contribute to bone erosion in RA. Indeed, increased levels of RANKL, which is also secreted by MCs, were found in the synovial tissue of RA patients (181, 182).

The specific role of MCs in RA was investigated in different mouse models of MC deficiency. However, these studies revealed contradictory results, depending on the MC-deficient mouse strain and the respective model of RA induction. Rivellese et al. recently reviewed these animal studies in detail (183). Briefly, Kit^W/W−v^ mice were protected from K/BxN serum-induced arthritis (serum from K/BxN mice contains autoantibodies against glucose-6-phosphate isomerase) (184, 185). However, Kit^W/Wv^ mice are fully susceptible to collagen-induced arthritis (CIA), which is induced by the injection of type II collagen in Freund's adjuvant (186). By contrast, another MC-deficient Kit^W−sh/W−sh^ mouse line developed arthritis induced by K/BxN serum as well as CIA (187, 188). However, as already mentioned, Kit-mutant mice exhibit severe alterations of the immune system beyond the MC-deficiency, which may possibly account for the inconsistent outcomes of these studies. Furthermore, the arthritis models used differ in their mechanisms of disease induction. In the CIA model, joint inflammation is induced by autoreactive effector T cells, while in the K/BxN model, immune cell infiltrations and activation is stimulated by the transferred autoantibodies thereby bypassing the T cell response (183).

c-Kit-independent MC-deficient Cpa3^Cre/+^ and Mcpt-5 Cre iDTR mice were not protected from K/BxN serum-induced arthritis (9, 118). However, Mcpt-5 Cre iDTR mice displayed reduced arthritis severity in CIA, indicating that MCs contribute to arthritis induction or progression by affecting the T cell arm of adaptive immunity (9, 118). Interestingly, in CIA, Mcpt-5 Cre iDTR mice showed reduced CD4+ and CD8+ T cell numbers in the lymph nodes draining the site of immunization accompanied by reduced IFN-γ and IL-17 production (Figure 1C). These results indicate that MCs may regulate T cell expansion and polarization to Th1 and Th17 effector cells in T cell-driven RA (9). Supporting these findings, the depletion of MCs during the early preclinical phase of CIA decreased joint inflammation in another model of c-Kit-independent inducible MC-deficiency, the RMB mouse. Similarly, numbers of CD4+ T cells, in particular IL-17 producing T cells, and serum levels of IL-6 and IL-17 were reduced also here (189). Additionally, in a pharmacological approach in wildtype mice, in which MCs were inhibited using salbutamol and cromolyn, RA development was diminished as indicated by reduced ankle swelling, joint inflammation, and bone destruction (185). Collectively, data of the CIA model demonstrate an important pro-inflammatory role of MCs in the onset of RA by promoting the expansion of autoreactive T cells and the T cell-driven inflammation (Figure 1C), whereas in the later disease phase, MCs may have redundant functions as implicated by most of the K/BxN studies. However, more studies are required to further decipher the specific role of MCs in RA-associated joint inflammation and bone resorption.

Interestingly, it has been shown that MCs may also play a role in OA, in which joint destruction is mainly caused by degeneration, abnormal high loads, or traumatic injuries (190), and driven by an increased inflammatory response (191). Several clinical studies reported increased MC numbers in the synovial tissue of OA patients and/or elevated histamine or tryptase levels in the synovial fluid (7, 192–196). Gene cluster analysis revealed increased expression of genes involved in MC differentiation and activity (*c-KIT*, tryptase genes *TPSAB1*, and *TPSAB2)* in the synovial membranes of OA patients (196). Interestingly, two different MC-deficient mouse lines, the c-Kit-dependent Kit^W−sh/W−sh^ line and the Kit-independent Cpa3^Cre^; Mcl-1^fl/fl^ mice, were protected from OA as demonstrated by reduced inflammation and cartilage destruction, while MC engraftment reversed the protective effects in both mouse lines (196). Furthermore, the inhibition of tryptase activity in wildtype mice prevented OA and reduced the concentrations of the pro-inflammatory and proteolytic mediators, e.g., IL-6, IL-1β, IL-8, and MMP-3. The authors further showed that in OA, MCs are activated via the IgE/FcεRI receptor axis (196). Another study showed that synovial MCs from OA patients produce TNF-α upon stimulation via the high-affinity receptor for IgG (174). These results indicate an important role of MCs in OA development. In support of these findings, a cross-sectional cohort study showed that the usage of H1anti-histamine treatment correlated with decreased OA prevalence (197), suggesting that MCs could potentially be a therapeutic target in OA, but this needs to be clarified in further studies.

### Bone Fracture Healing

The immune system plays a major role in bone repair, because the healing process begins with an acute immune response locally at the fracture site (207, 208). In addition, conditions of acute orresults indicate that MCs may regul chronic inflammation, including poly-trauma, osteoporosis, and RA, negatively impact the fracture healing outcome (207). Bone fracture leads to the rupture of blood vessels and to tissue and cell damage, resulting in the formation of a hematoma, which is characterized by hypoxia, low pH, high lactate levels, as well as high concentrations of inflammatory mediators that attract cells of the innate immune response. First, neutrophils invade the fracture hematoma. They secrete further cytokines, including chemokine (C-X-C motif) ligand 1 (CXCL1) and IL-1β, which attract other immune cells, mainly macrophages. These cells further phagocytize cell and tissue debris and pathogens. Subsequently, T and B cells arrive and initiate adaptive immune responses. Consequently, angiogenesis starts, ensuring debris removal, nutrient and oxygen supply, and the recruitment of mesenchymal stem cells (MSCs). Recruited MCSs initiate the repair phase, where in the process of endochondral healing, first a cartilaginous soft callus is generated that is converted into a hard bony trabecular callus. The bony callus is finally remodeled to the original bone shape (207, 209). Studies have shown that certain immune cell populations, including neutrophils, macrophages, but also B and T cells, essentially contribute to successful bone repair, because their absence or disturbed function resulted in disrupted fracture healing (210–212). This might also be true for MCs. Indeed, some older phenomenological studies described MC appearance in bone repair, while more recent studies using different MC-deficient models also revealed some specific MC functions. The few existing studies that explored MCs in fracture healing are summarized in Table 2.

**Table 2:** Experimental studies investigating MC appearance and function in fracture healing.

**References**	**Model**	**Treatment**	**Main results**
Lindholm et al. (198)	White rats (m/f), tibia fracture	–	Progressive MC accumulation in the periosteal callus; MCs decreased during callus remodeling
Lindholm et al. (199)	White rats (m/f), tibia fracture	17-hydroxy-corticosterone	Progressive MC accumulation in the periosteal callus; delayed healing due to treatment affected MC morphology in size, granulation, and staining
Lindholm et al. (200)	White rats (m/f), tibia fracture	Somatotropin and thyrotropin	Progressive MC accumulation in the periostal callus; improved healing due to treatment led to earlier MC accumulation
Lindholm and Lindholm (201)	Rabbits (m/f), forearm fracture	–	MCs more abundant in the periosteal callus compared to the endosteal callus
Taniguchi (41)	Wistar rats (m), bilateral tibia fracture	–	Few MCs near blood vessels and in the marrow of the early endosteal callus; MCs increase in the late periosteal callus and peak during remodeling
Banovac et al. (202)	Sprague-Dawley rats (f), femur fracture	NSAIDs	Few MCs near blood vessels and cartilage of the early endosteal callus; MC accumulation near osteoclastic bone resorption during late remodeling; NSAIDs delayed healing and MC appearance
Meyer et al. (203)	Sprague-Dawley rats (f), femur fracture	–	Microarray analysis revealed increased MC marker activity from weeks 2 to 4 after fracture in all age groups
Behrends et al. (11)	MC-deficient Kit^W−sh/W−sh^, C57BL/6J mice, femoral cortical window defect	–	Disturbed healing in MC-deficient mice: ↓ Cortical bridging, bone content, endothelial cells, macrophages; ↑ TRAP+ cells
Ramirez-GarciaLuna et al. (204)	MC-deficient Cpa3^Cre/+^ and C57BL/6J mice (m/f), femoral cortical window defect	–	MCs appeared in the connective tissue and marrow of the defect; Disturbed healing in MC-deficient mice: ↓ Cortical bridging, bone content, vascularization, bone mineralization, osteoclasts
Kroner et al. (10)	MC-deficient Mcpt-5 Cre+/– R-DTA mice (m), femur fracture	–	MCs increase in the periostal callus near newly formed bony trabeculae and osteoclastic bone resorption sites; Disturbed healing in MC-deficient mice: ↓ Local and systemic inflammation (cytokine release, immune cell recruitment); ↑ bone content; ↓ osteoclastic remodeling
Zhang et al. (205)	C57BL/6J, Mcpt-5 Cre YFP, Mcpt-5 Cre iDTR mice (m/f), cranial window defect	rPTH, SC	MC inhibition (SC) and deficiency (Mcpt-5 Cre iDTR): ↑ Healing, ↓ arteriogenesis; rPTH: ↓ MCs in the inflammatory phase by acting on osteoblasts releasing anti-MC factors
Hebb et al. (206)	C57BL/6 (m/f), bilateral tibia fracture	–	Microarray analysis revealed increased MC prevalence in younger compared to older mice

The presence of MCs during fracture healing was already described in 1967 by the group of Lindholm using a rat tibial fracture model. The authors showed that MC numbers progressively increase in the periostal fracture callus, followed by a decline during callus remodeling (198). In further investigations of experimentally delayed or accelerated fracture healing, the same group observed alterations in MC accumulation, morphology, and degranulation (199, 200). On the basis of these results, the authors concluded that MC invasion and degranulation are essential for endochondral bone formation and mineralization. The presence of MCs in the periostal callus was further confirmed in a rabbit fracture model (201). Two later studies confirmed MC accumulation during fracture healing and both described only a few MCs during the early healing phase, mainly around blood vessels and in the bone marrow cavity of the endosteal callus (41, 202). In the later healing phases, increasing MC numbers were observed in the marrow of the newly formed periostal callus, particularly next to newly formed bony trabeculae. Both studies found the highest numbers of MCs in close proximity to osteoclasts and bone resorption sites during the callus-remodeling phase and suggested that MCs might contribute to callus remodeling by influencing osteoclast activity (41, 202). Furthermore, microarray analysis of a rat femoral fracture callus found increased MC marker gene expression, including the MC *tryptase* β*1* and *Cpa3* from weeks 2 to 4 after fracture (203). In addition, a recent microarray analysis of a tibial fracture callus of young and old C57Bl/6 mice found a higher MC occurrence in younger mice compared to old mice evaluated by cell type enrichment analysis, showing higher MC *IgE* gene expression in young mice (206). On the basis of the above-mentioned studies, the presence and accumulation of MC is the fracture callus is clear (Figure 1D).

To elucidate MC functions, Behrends et al. investigated bone repair in an uni-cortical window defect of MC-deficient Kit^W−sh/W−sh^ mice (11). Interestingly, MC-deficient Kit^W−sh/W−sh^ mice displayed a delayed healing with reduced bone quality because of an impaired transformation of woven into lamellar bone. The authors further observed diminished endothelial cell numbers, but increased numbers of osteoclasts, and suggested that healing was impaired because of disturbed revascularization and increased osteocatabolic activity (11). However, these results were obtained in a c-Kit-dependent mouse model, from which it is known that also osteoclasts and other immune cells are affected (113, 115). This could have influenced the outcome. Investigations of bone repair in a cortical window defect in a c-Kit-independent Cpa3^Cre/+^ mouse model found impaired bone regeneration, as confirmed by reduced cortical bridging, vascularization, and bone mineralization. The authors further observed diminished osteoclast activity at earlier stages, but increased osteoclast activity in the late healing phase (204). Therefore, MC functions in bone repair may comprise blood vessel formation as well as anabolic and catabolic processes during fracture repair and remodeling (Figure 1D). However, it was shown that Cpa3 is also expressed in basophils and some T cells (109), which needs to be considered when interpreting these results. Overcoming these drawbacks, our group recently investigated the functions of MCs in bone repair in MC-deficient Mcpt-5 Cre R-DTA mice, which lack CTMCs without affecting other immune cell populations (116, 213). Interestingly, we found reduced levels of pro-inflammatory cytokines, including IL-6, IL-1β, and CXCL1, locally in the early fracture callus, but also systemically, and a reduced recruitment of neutrophils and macrophages to the fracture site in the absence of MCs (10). These results indicate a strong contribution of MCs to fracture-induced systemic inflammation and to the inflammatory mediator and cell milieu at the fracture site (Figure 1D). MC-mediated neutrophil recruitment was already described during acute inflammation, including bacteria-induced pneumonia and ischemic-induced gut injury, as well as in inflammatory diseases such as meningitis and periodontitis, contributing to the disease onset and progression (214, 215). During the later healing stage, we found an increased bone content of the fracture callus in MC-deficient Mcpt-5 Cre R-DTA mice (10). Further histomorphometric analysis revealed no changes in osteoblast parameters, however, osteoclast numbers and activity were significantly reduced in the fracture callus of Mcpt-5 Cre R-DTA mice (10). These results indicate that MCs may mediate callus remodeling by regulating osteoclast activity (Figure 1D). As indicated earlier in the osteoporosis chapter, MC-derived histamine might be one mechanism contributing to increased osteoclastic bone resorption. Supporting our experimental outcomes, Zhang et al. investigated bone repair in a cranial window defect model in MC-deficient Mcpt-5 Cre iDTA mice, and found accelerated defect closure and impaired angiogenesis in the absence of MCs (205). They observed the same effects by inhibiting MCs in wildtype mice using cromolyn, and suggested that MCs may be negative regulators of bone repair (205).

Concluding, several studies demonstrated MC accumulation in the periostal fracture callus during the healing process. More recent experimental studies also revealed possible functions of MCs in fracture healing, including the regulation of the immune response toward fracture and of angiogenesis as well as anabolic and catabolic effects during the repair and remodeling processes (Figure 1D).

## Conclusion

The important role of MCs in allergic reactions has been known for several decades. However, the involvement of MCs in physiological bone turnover and bone disorders has been described only recently in more detail. As reviewed here, MCs secrete several mediators that are known to regulate bone formation and resorption, including histamine, IL-6, and TNF. Experimental data on the role of MCs in physiological bone turnover are contradictory and depend on the mouse model used. However, the involvement of MCs in various pathological skeletal conditions is clear, particularly in osteoporosis and RA. MCs may also regulate the fracture healing process by influencing the inflammatory response, angiogenesis, bone formation, and osteoclastogenesis (Figure 1). Osteoclastogenesis might be mainly, but not solely, regulated by MC-derived histamine. Further mechanistic investigations are required to elucidate MC functions in physiological and pathological conditions in bone. In consequence of the involvement of MCs in bone disorders, MC targeting drugs such as histamine H1 receptor blockers should be further tested for their therapeutic potential to treat osteoporosis, inflammatory bone disorders or disturbed bone repair.

## Author Contributions

All authors listed have made a substantial, direct and intellectual contribution to the work, and approved it for publication.

### Conflict of Interest

The authors declare that the research was conducted in the absence of any commercial or financial relationships that could be construed as a potential conflict of interest.

## Abbreviations

CIA: Collagen-Induced Arthritis
Cpa3: Carboxypeptidase A3
CTMCs: Connective Tissue Mast Cells
CXCL: Chemokine (C-X-C Motif) Ligand
DT: Diphtheria Toxin
DTR: Diphtheria Toxin Receptor
ER: Estrogen Receptor
f: female
FcεRI: High Affinity Immunoglobulin E (IgE) Receptor
FGF: Fibroblast Growth Factor
GM-CSF: Granulocyte-Macrophage Colony-Stimulating Factor
IL: Interleukin
INF-γ: Interferon-γ
m: male
Mas-TRECK: Mast Cell-Specific Enhancer Mediated Toxin Receptor Mediated Conditional Cell Knockout
MCs: Mast Cells
Mcl-1: Myeloid Cell Leukemia Sequence-1
MCP-1: Monocyte Chemoattractant Protein-1
MCps: Mast Cell Progenitors
Mcpt: Mast Cell Protease
M-CSF: Macrophage Colony-Stimulating Factor
MIP-1α: Macrophage Inflammatory Protein-1α
MMCs: Mucosal Mast Cells
MMPs: Matrix Metalloproteinases
MSCs: Mesenchymal Stem Cells
NO: Nitric Oxide
NSAID: Non-Steroidal Anti-Inflammatory Drugs
OA: Osteoarthritis
OVX: Ovariectomy
PAF: Platelet Activating Factor
RA: Rheumatoid Arthritis
RANKL: Receptor Activator Of Nuclear Factor Kappa B Ligand
RFP: Red Fluorescent Protein
RMB: Red Mast Cell And Basophil Mouse
rPTH: Teriparatide
SC: Sodium Cromolyn
SCF: Stem Cell Factor
SM: Systemic Mastocytosis
tdT: td-Tomato
TGF-β: Transforming Growth Factor-β
TLR: Toll-Like Receptor
TNF: Tumor Necrosis Factor
TRAP: Tartrate Resistant Acid Phosphatase
VEGF: Vascular Endothelial Growth Factor
YFP: Yellow Fluorescent Protein
↓: Decreased
↑: Increased.
